# Knowledge and Attitudes on Vaccination in Southern Romanians: A Cross-Sectional Questionnaire

**DOI:** 10.3390/vaccines8040774

**Published:** 2020-12-18

**Authors:** Gabriela Loredana Popa, Andrei-Alexandru Muntean, Mădălina-Maria Muntean, Mircea Ioan Popa

**Affiliations:** 1 Department of Microbiology, Faculty of Dental Medicine, The “Carol Davila” University of Medicine and Pharmacy, 050474 Bucharest, Romania; gabriela.popa@umfcd.ro; 2 Department of Microbiology II, Faculty of General Medicine, The “Carol Davila” University of Medicine and Pharmacy, 050474 Bucharest, Romania; alexandru.muntean@umfcd.ro (A.-A.M.); madalina.muntean@drd.umfcd.ro (M.-M.M.); 3 The “Cantacuzino” National Medico-Military Institute for Research and Development, 050096 Bucharest, Romania

**Keywords:** vaccines, questionnaire, knowledge and attitudes

## Abstract

Vaccines are fundamental instruments upon which all modern medicine is hinged. This has recently come into the light because of the COVID-19 pandemic caused by SARS-CoV-2. We aimed to assess the knowledge and attitudes of the public regarding vaccination. To this end, a questionnaire, which was disseminated to the general population between 2017 and 2019, was used. We evaluated the responses from 1647 individuals (61% female, with a median age of 37 years, mostly from urban settings). Most respondents (85%) had children and were in favor of vaccination. Our study underlines the role that family physicians have in the education and information of citizens. A small, but considerable, number of respondents (108, 7.84% of those with children) had not vaccinated their children according to the national vaccination scheme. Deterrents were considered to be lack of information and fear of side effects. However, 167 of our respondents (12.12% out of 1377 respondents with children) said that their child experienced adverse events—most of which were mild local reactions. Alternatives to vaccination were proposed by some. In this study, we highlight the attitudes of respondents and multiple gaps in general knowledge, both of which may need to be addressed, especially in light of the current pandemic situation and past failed campaigns.

## 1. Introduction

In the context of a pandemic that, 10 months in, still includes many unknowns [1,2,3,4], bearing in mind the high expectations for the production and provision of a vaccine [5,6,7,8], it is very important to know how mass vaccination would be viewed and, as much as possible, identify what should be done to make vaccine prevention of COVID-19 possible. Vaccination initiatives may be hindered by antivaccination movements, which leads to a series of difficulties [9,10,11,12,13]. At the same time, lockdown fatigue leads to waning trust in the authorities, and the restrictions imposed lead to sometimes violent reactions.

We aimed to assess the knowledge and attitudes regarding vaccination in relation to the perception of the risks of the most cost-effective preventive method known and applied a questionnaire to 1647 respondents from several counties in our country.

## 2. Materials and Methods

The questionnaire was administered between 2017 and 2019 by medical students and residents from the “Carol Davila” University of Medicine and Pharmacy. The questionnaire was peer validated by the National Institute for Public Health and approved by the Ethics Committee of the “Carol Davila” University (123/24.03.2017). The questionnaire (which may be found in Supplementary Material 1) is composed of 41 data points (some of which were yes/no questions while others were free-form answers) and was printed on a single piece of paper and handed to medical students and residents who administered it to willing participants. No personal information was solicited from the participants. The questionnaire was applied, when possible, in a direct interview; as an alternative, telephone interviews or World Wide Web resources were used. Data were collected in EpiInfo (v. 7.2) (Communicable Disease Center, Atlanta, GA, USA) and analyzed using R (v. 4.0.2) ( (R Foundation for Statistical Computing, Vienna, Austria).

## 3. Results

In total, 1647 individual answers were received. Responses to questions are summarized in Supplementary Material 2.

Participants had a median age of 37 (interquartile range: 15; min–max: 16–77) and were mostly female (61%). Urban respondents were the most frequent (70%, 1149). Three-quarters of all respondents were from nine counties: Bucharest, Prahova, Argeș, Constanța, Galați, Vâlcea, Ilfov, Călărași and Buzău, all counties from southern Romania. Characteristics of the respondents are summarized in Table 1.

Of the total participants in our study, 1377 (85%) had children. Most had one child (738, 52%), with a wide variability range (interquartile range: 1; min–max: 0–10 children).

When asked if they know the benefits of vaccines, most respondents answered that they did; however, 80 respondents (4.85%) said that they did not know the benefits, 40 were unsure of the validity of the information they had (2.42%) and 16 (almost 1%) failed to respond and could not be reached for further follow-up.

Most participants said that they consulted more than one source of information about vaccines. However, about one-third (32%) of the respondents said that they had received information about vaccine effectiveness from only one source. In both cases, the family physician (76%) was the most important information source, followed by Internet sources (45%), family (31%), friends (28%), literature (25%) and other sources (12%).

When questioned about six childhood vaccines administered in Romania, participants recognized a median of five of them. Interestingly, while 42% of respondents identified all vaccines, nearly 10% could only identify one (2.8%) or two (7%) vaccines.

With regards to the most recognizable vaccines, the anti-HBV vaccine was the most often recognized (80.6%), followed by the measles (77.8%), polio (72.8%), rubella (72.3%), DTP (68.7%) and, lastly, the BCG (67%) vaccines.

Respondents were further asked if their child was fully immunized according to the current Romanian vaccination calendar. Only a minority of respondents said that their children are not fully vaccinated according to the national scheme (*n* = 108, 7.84% of the 1377 respondents with children).

When asked what would constitute a deterrent to vaccination, lack of information (77%) and fear of adverse reactions (60.35%) were considered top deterrents, while price was less influential (20.46%).

Further information was offered by 93 respondents. Disinformation (through classic media, social media and the Internet) was considered a deterrent by 25 respondents, closely followed by fears of toxicity and poor quality related to vaccine components and doubts about the technology used to produce the vaccine—23 respondents. Personal reasons to refuse vaccines (which included religious conviction) were the third deterrent mentioned (*n* = 17). Lack of trust in the healthcare system, specific education and the influence of antivaccine proponents (“antivaxxers”) were all reported to a lesser degree.

One hundred seventy-six respondents (12.12% out of 1377 respondents with children) said that their child experienced adverse events. No information on the nature of the adverse event was mentioned in 32 cases. Of those who gave details, most reported fever (101 cases), local swelling/pain, nausea/vomiting, systemic side effects (drowsiness, agitation or apathy) or mild allergic reactions. Serious side effects were rarely reported by parents and included postvaccination illness (measles and rubella), poliovirus sequelae, convulsions and even one death (although it is very difficult to link this tragic event to vaccination).

Even though most people have vaccinated their children, the respondents of our study were not in favor of introducing sanctions to enforce immunization (48.6%). While almost half of the respondents were definitely against sanctions, one-quarter of the respondents were either in favor of sanctions (24.8%) or unsure if sanctions would be beneficial (23.4%). Breakdown according to sex and educational level yielded no statistically significant differences (Fisher’s exact test, *p* > 0.5).

Respondents who answered in favor of sanctions were asked what kind of sanctions they would impose—253 detailed their answers. Most were in favor of economic sanctions (60% of 253 answers), either as fines or as payment of the healthcare costs of people who may get sick. Other responses included limiting access of unvaccinated children to public kindergartens and schools (17%), placing personal sanctions on the parents (7.5%) and establishing a more clear law on vaccination (3.9%).

## 4. Discussion

The importance of knowledge of and attitudes towards vaccines and vaccination has become central in the past few months. The lack of a vaccine for just one communicable disease (COVID-19) has profoundly impacted social and economic life and has led to the deaths of hundreds of thousands [14,15]. While reported vaccine candidates are promising options, the need for high coverage and the existence of a hesitant population may hamper efforts. Our study investigated knowledge of and attitudes towards vaccination in a population of 1647 respondents, mainly from southern Romania. While this is a limited sample size, these data may be considered representative.

Most respondents were younger urban parents, who would have been directly interested in the vaccination of their children. Some were elderly respondents, who may not have been aware of current vaccination indications for seniors.

The main conclusion of our study is that most of our respondents trust their family physician for getting their information about vaccines and vaccination. This is in contrast to past data, where the role of family physicians seemed to be on the downfall. Data collected before 2017 showed that less than 60% of respondents had information from family physicians and that over 80% believed that vaccines could be refused due to side effects. This is in contrast to under 30% of respondents reporting, from their personal experience, mild and moderate side effects from vaccination [16]. This is important to know, as family physicians need to undergo continuous medical education and offer vaccination counseling to all patients.

Regarding vaccination deterrents, lack of clear, constructive information and fear of side effects played the biggest role. Even so, respondents who have vaccinated their children reported having experienced few vaccine-related side effects. The rates are comparable to other published studies. Price seems to play a small role as the vaccines included in the national immunization program are offered free of charge to the population. However, some physicians recommend vaccines that are not covered by the national vaccination program. In recent years, the immunization calendar has been updated to include the conjugated pneumococcal vaccine and the *Haemophilus influenzae* type b vaccine. Along with these, there are many optional vaccines that are available on the market (meningococcal vaccines, hepatitis A vaccine, etc.).

Up until 2009, Romania produced vaccines at the Cantacuzino National Institute. Interestingly, some respondents said that vaccines produced outside the country are not trustworthy and that they would only get vaccinated if the vaccine was produced by the Cantacuzino Institute. Even regarding the appearance of a SARS-CoV-2 vaccine, Romanians would more easily accept the vaccination if the vaccine were produced in Romania (as reported by multiple media outlets).

The small number of people that expressed antivaccine conviction among our respondents suggests we had a selection bias. Indeed, the number of people who have antivaccine sentiments in Romania has increased, as can be seen through the ongoing measles outbreak. In 2014, the measles vaccine coverage dropped below 90% [17].

Efforts to introduce new vaccines have been met with resistance, as was the case with the HPV vaccine’s introduction in 2007. Romania had initiated a program in 2008 but discontinued their HPV vaccination in 2014 due to a very low acceptance rate [18]. The HPV vaccination campaign was a major failure. The campaign started without following the most basic rules and without preparing the population. That campaign left consequences that have effects even today. When comparing the current situation with the 1998–1999 period, when we organized the largest nationwide vaccination campaign, vaccinating about 2.1 million people without serious side effects and without refusals to vaccinate, the problems related to accepting vaccination are significant [19]. The phenomenon of vaccine refusal has emerged as a serious social movement and is spreading worldwide [20,21,22,23,24,25]. The problem is critical and worrisome. The current pandemic, with all of its implications and unknowns, must awaken the whole of society to a better current and future management of similar crises. Only nine years have passed since the previous pandemic. Unfortunately, no major, positive changes were implemented after 2009.

Social or traditional media may choose to present information according to their own interests, or as a reflection of their understanding of the matter. An analysis of the failed HPV vaccination campaign in Romania has shown that media coverage in the first year was mostly negative [26]. While this is true for any subject that may come under media scrutiny, the presence of mixed information (both true and misleading) within the same article may sway readers to draw a conclusion. Some Internet sites, which are unregulated and are not affiliated with any type of media, may choose to present outright propaganda [27].

Some respondents were in favor of enforcing sanctions. Governing bodies could benefit from a look at history. Past examples of vaccine enforcement, refusal and penalty may offer more information and guide policy.

As alternatives to vaccines, respondents preferred general “natural” methods. Some cited isolation, mask-wearing and rigorous hygiene as alternatives. While these are general preventive measures, the lockdown caused by the SARS-CoV-2 pandemic and the ensuing fatigue prove that these measures work only for a limited amount of time before negatively impacting society [28]. A small number of respondents (*n* = 3) mentioned antibiotics as an alternative to vaccination. While antibiotics and vaccines mostly have very different indications, use of antibiotics during the COVID-19 pandemic should be expected to increase levels of antimicrobial resistance in the medical, veterinary and environmental sectors [29].

The study suffered from selection bias due to the subjects being selected according to the possibilities of those who applied the questionnaires and being mainly located in the south of Romania. The study cannot be extrapolated to be relevant at a national level. Our intention is to update and refine the questionnaire and the deployment methods, and we intend to collaborate with teams from other university centers in the coming year. The open-ended questions did not lead to useful information, but analysis of open responses may improve future questionnaire development.

Even if our intention was to reconnect with each respondent in order to clarify any ambiguities, answer their questions and disseminate the correct information, this was not possible in the current format, and this activity would be resumed after the pandemic.

## 5. Conclusions

A discussion about a Romanian vaccination law has been ongoing for about 10 years—a project was developed 3 years ago. Its legislation is still unsure. Even if it is approved, the law will not solve all of the problems accumulated in the last 12 years, especially after the failure to implement the anti-HPV program. In our opinion, the situation cannot be improved without regaining the confidence of the population.

Attitudes observed in current medical practice, media articles and the results of our study demonstrate an acute need for correct information. The vectors of this important communication must be unanimously recognized specialists.

Family physicians need to be reinstated as partners in public health, and their work should be freed of bureaucracy. They have the greatest possibility of access (medical, social, psychological, etc.) to the families they care for. All physicians should think about and heed the words of Plotkin and Mortimer in the foreword of the first edition of their seminal textbook *Vaccines*: “The cure is more interesting than prevention. Minute attention is focused on the heart transplant, the surgery of separating Siamese twins, the high technology of intensive care and why not? These are where the drama is perceived to be. And yet no single physician can in his or her career hope to save more than a small fraction of the lives saved by a single vaccine.” [30].

## Figures and Tables

**Table 1:** Sociodemographic and educational characteristics of respondents to the questionnaire.

Characteristics	*N* = 1647
Are parents	1377 (85%)
Sex (Women)	1002 (61%)
Age (median, min-max)	37, 16–77
Geographic area (Urban)	1149 (70%)
**Studies**	
None	9 (0.5%)
Primary School	19 (1.2%)
Secondary School	429 (26%)
High School	79 (4.6%)
College	1114 (68%)

## Supplementary Materials

The following are available online at https://www.mdpi.com/2076-393X/8/4/774/s1, Supplementary Material 1: Vaccination Questionnaire; Supplementary Material 2: Questionnaire Response.
