# Supplementary material for: Knowledge and Attitudes on Vaccination in Southern Romanians: A Cross-Sectional Questionnaire
Source: Vaccines (Basel). 2020 Dec 18;8(4):774. doi: 10.3390/vaccines8040774 (PMC7765821; doi:10.3390/vaccines8040774)
Supplement: Supplementary file 1 [file vaccines-08-00774-s001.zip › vaccines-1003851 supplementary proof/Supplimentary_material_1_Vaccine_questionnaire.pdf]

Vaccination Questionnaire

1. Do you have children? Yes No /

2. Number of children …. **3**. Sex M / F….

4. How old are you (years old)? .... years **5**. R / U residential environment

6 .County ……………………… **7** .Occupation ………………………………………………………

8 .Level of education without / primary / secondary / highschool / higher education **9**.Other education …………………

Questions	Yes	Not	I do not know
**Do you know the benefits of vaccinating children?**			
**If "Yes", from which sources?**			
General Physician (Family physician)			
Internet			
Family			
Friends / acquaintances			
Specialty literature			
Other			
**Do you think vaccines are risky?**			
**What vaccines are given to children in Romania?**			
BCG			
Against hepatitis B			
Against diphtheria-tetanus-whooping cough			
Against measles			
Against rubella			
Against polio			
**What do you think are the reasons why parents do not agree with certain vaccines** ?			
Lack of correct information about the vaccine			
Price			
Side effects			
Other reasons …………………………………………………………………			
**Has your child/children been vaccinated according to the Romanian vaccination schedule?**			
**Have any side effects occurred as a result of vaccinating your child / children?**			
If "Yes", can you give an example?			
**Have you ever been told by your doctor that vaccination is absolutely necessary?**			
**Do you think that there should be legal sanctions / constraints in case of parents' refusal to vaccinate their children?**			
If "Yes" please give an example.			
**What alternatives to vaccination do you think would be helpful in preventing your child / children from getting sick?**			

Thank you for the kindness with which you answered our questions and for the time you gave us!

National Institute for Public Health—National Center for Surveillance and Control of Communicable Diseases & "the Carol Davila" University of Medicine and Pharmacy, Bucharest
