# Supplementary material for: Knowledge and Attitudes on Vaccination in Southern Romanians: A Cross-Sectional Questionnaire
Source: Vaccines (Basel). 2020 Dec 18;8(4):774. doi: 10.3390/vaccines8040774 (PMC7765821; doi:10.3390/vaccines8040774)
Supplement: Supplementary file 1 [file vaccines-08-00774-s001.zip › vaccines-1003851 supplementary proof/Supplimentary_material_2_Questionnaire_response.pdf]

Questionnaire Response

Characteristic	*N* = 1647			
**Do you have children? (Yes)**	1377 (85%)			
Unknown/No response	23			
**Number of children**				
0	247 (15%)			
1	738 (52%)			
2	502 (35%)			
3	84 (5.9%)			
4	17 (1.2%)			
5	10 (0.7%)			
6	3 (0.2%)			
10	1 (<0.1%)			
Unknown/No response	48			
**Sex**				
Feminine	1002 (61%)			
Masculine	555 (34%)			
Unknown/No response	90 (5.5%)			
**Age**	37 (IQR: 30, 45)			
Unknown/No response	54			
**Mediu**				
Urban	1149 (70%)			
Rural	425 (26%)			
Unknown/No response	73 (4.4%)			
**Studies**				
None	9 (0.5%)			
Primary School	19 (1.2%)			
Secondary School	429 (26%)			
High School	79 (4.6%)			
College	1114 (68%)			
**Question**	**Yes**	**No**	**I don’t know**	**Unknown/No response**
**Do you know the benefits of vaccinating children?**	1511 (92%)	80 (4.9%)	40 (2.4%)	16 (1.0%)
**If "Yes", from which sources?**				
General Physician (Family physician)	1257 (76%)	228 (14%)	7 (0.4%)	155 (9.4%)

Internet	733 (45%)	635 (39%)	11 (0.7%)	268 (16%)
Family	511 (31%)	795 (48%)	15 (0.9%)	326 (20%)
Friends / acquaintances	454 (28%)	864 (52%)	14 (0.9%)	315 (19%)
Specialty literature	404 (25%)	895 (54%)	22 (1.3%)	326 (20%)
Other	204 (12%)	952 (58%)	33 (2.0%)	458 (28%)
**Do you think vaccines are risky?**	805 (49%)	499 (30%)	293 (18%)	50 (3.0%)
**What vaccines are given to children in Romania? (Yes)**				
BCG	1103 (84%)			
VHB	1329 (91%)			
DTP	1131 (83%)			
Measles	1283 (87%)			
Rubella	1191 (83%)			
Polio	1199 (89%)			
**What do you think are the reasons parents do not agree with certain vaccines? (Yes)**				
Lack of correct information	1192 (83%)			
Price	337 (30%)			
Side effects	994 (73%)			
**Has your child/children been vaccinated according to the Romanian vaccination schedule?**	1269 (89%)	108 (7.6%)	47 (3.3%)	223 (13.5%)
**Have any side effects occurred as a result of vaccinating your child / children?**	177 (11%)	1169 (71%)	59 (3.6%)	242 (15%)
**Have you ever been told by your doctor that vaccination is absolutely necessary?**	1227 (74%)	306 (19%)	63 (3.8%)	51 (3.1%)
**Do you think that there should be legal sanctions / constraints in case of parents' refusal to vaccinate their children?**	409 (25%)	800 (49%)	386 (23%)	52 (3.2%)
